# Epigenome-wide association study of psilocybin-induced methylome changes in alcohol use disorder

**DOI:** 10.1038/s41398-026-03961-3

**Published:** 2026-05-26

**Authors:** Marvin M. Urban, Lea Zillich, Nathalie M. Rieser, Marcus Herdener, Rainer Spanagel, Franz X. Vollenweider, Katrin H. Preller, Marcus W. Meinhardt

**Affiliations:** 1 Institute of Psychopharmacology, Central Institute of Mental Health, Medical Faculty Mannheim, University of Heidelberg, Mannheim, Germany; 2 Interdisciplinary Center for Neurosciences, University of Heidelberg, Heidelberg, Germany; 3 Department of Psychiatry and Psychotherapy, University Medical Center Freiburg, Freiburg, Germany; 4 Department of Genetic Epidemiology in Psychiatry, Central Institute of Mental Health, Medical Faculty Mannheim, University of Heidelberg, Mannheim, Germany; 5 Department of Adult Psychiatry and Psychotherapy, Psychiatric University Clinic Zurich and University of Zurich, Zurich, Switzerland; 6 Department of Molecular Neuroimaging, Central Institute of Mental Health, Medical Faculty Mannheim, University of Heidelberg, Mannheim, Germany

**Keywords:** Molecular neuroscience, Addiction

## Abstract

The serotonergic hallucinogen psilocybin has shown potential as a treatment for psychiatric conditions like alcohol use disorder (AUD) and depression in clinical studies. Epigenetic mechanisms, including DNA methylation, are hypothesized to contribute to its lasting therapeutic benefits. In this exploratory study, we present the first methylome-wide analysis of psilocybin-induced changes in a cohort of detoxified patients with AUD. The longitudinal study design included three assessment days in 37 patients with blood sampling and acquisition of psychometrics – at baseline, 24 h after administration of psilocybin (25 mg) or placebo (mannitol), and one month after treatment. As the primary endpoints (duration of abstinence and mean alcohol use) in this trial were not reached, our investigation included secondary psychometrics that differed significantly between groups: Beck’s Depression Inventory and Beck’s Hopelessness Scale. The epigenome-wide association study (EWAS) identified one CpG site in *TLE4* (*p* = 1.1e-7) associated with psilocybin treatment. Screening for differentially methylated regions, we observed altered methylation in the gene *RASGRP4* (*pFDR* = 3.2e-4). Network analysis revealed co-methylation modules related to psilocybin treatment, as well as modules associated with the reduction of depressive symptoms and drinking behavior. Gene ontology analysis indicated involvement of these modules in neuroplasticity and immune functions, suggesting that they may reflect abstinence-related recovery processes. Investigating candidate genes at nominal significance (*p* < 0.05) uncovered promoter-associated methylation changes in *HTR2A* and *TNF*. Interestingly, several of the reported analyses point to immunomodulatory actions of psilocybin. While the findings of this pilot study are limited by the modest sample size, they align well with previous literature and might provide starting points for further, large-scale investigations or hypothesis-driven experiments.

## Introduction

Alcohol use disorder (AUD) contributes significantly to the global disease burden, with more than 5% of annual deaths being attributable to excessive alcohol use [1]. Current approved pharmacotherapies for AUD, like acamprosate, disulfiram, or naltrexone, show modest treatment success and require regular dosing, which impedes adherence to therapeutic protocols [2–4].

Contrasting the dosing regimen of established medications for AUD, psychedelic compounds, such as psilocybin, may be able to induce lasting reductions in alcohol use after a single or few administrations [5–7]. While the underlying mechanisms are not fully understood, recent neurobiological findings suggest a cascade of biological effects involving alterations in gene expression, induction of neuronal plasticity, and changes in functional network connectivity that facilitate shifts in cognition and behavior [8–12]. This chain of effects might also include the level of epigenetics [13, 14].

Several studies support the idea of epigenetic changes in psychedelic drug action. Evidence from preclinical experiments points towards changes in histone acetylation after administration of lysergic acid diethylamide (LSD) [15] and 2,5-dimethoxy-4-iodoamphetamine (DOI) [16]. Furthermore, a genome-wide methylation analysis after repeated LSD administration revealed 635 differentially methylated cytosine-guanine dinucleotides (CpG sites) in the prefrontal cortex of mice [17]. In a naturalistic human study on ayahuasca, which contains the psychedelic dimethyltryptamine (DMT), increased methylation across five CpG sites in the promoter region of the sigma-1 receptor was found [18].

Blood DNA methylation has been proposed as a biomarker to predict treatment responses to pharmacological treatments [19, 20] and presents a more feasible and scalable target for clinical investigation than neuronal tissue. For instance, although some of the evidence is only suggestive, genome-wide methylation patterns measured before pharmacotherapy with antidepressants were associated with treatment success in several studies [21–23].

Here we present the first epigenome-wide association study (EWAS) of psilocybin in patients with AUD. Specifically, we investigated longitudinal changes in blood DNA methylation in response to psilocybin treatment in a sample of detoxified patients (*n* = 37). The methylation and psychometric data presented here stem from a randomized clinical trial (RCT) that investigated the effect of psilocybin on alcohol relapse and abstinence. This RCT was conducted at the Psychiatric University Hospital in Zurich, Switzerland [24]. While the primary outcomes at 4-week follow-up did not differ between the placebo and psilocybin group – duration of abstinence and mean alcohol use – secondary clinical endpoints, such as depressive symptoms and quality of life, improved significantly in the psilocybin group. This provides a rationale to explore underlying epigenetic correlates, given the frequent comorbidity of depression and AUD [25, 26]. In the here presented exploratory analysis, we hypothesized i) associations between psilocybin treatment and methylation changes, ii) a mediating effect of methylation changes on depressive symptom reduction in the AUD cohort, and iii) differences in methylation patterns between responders (abstinent at 4-week follow-up) and non-responders to psilocybin treatment. We tested these hypotheses in a methylome-wide manner and for a selection of candidate genes. In our analyses, we distinguish between the following potentially overlapping, but distinct types of effects: i) psilocybin effects in general, referring to methylomic signatures induced by the substance without relation to psychometric changes; ii) markers of treatment response, describing methylomic differences that predict psychometric responses to psilocybin either at baseline or after administration; iii) putative mechanistic mediators, i.e. psilocybin-induced methylome changes that may causally affect symptom load; and iv) molecular correlates of symptom change and abstinence that may be unrelated to the drug intervention.

## Materials and methods

### Study design, participants, & psychometrics

This research is based on the study Clinical and Mechanistic Effects of Psilocybin in Alcohol Addicted Patients (clinicaltrials.gov identifier: NCT04141501; Kofam identifier: SNCTP000003445) conducted at the Psychiatric University Hospital in Zürich, Switzerland, by Rieser et al., [24]. The study was a randomized, placebo-controlled, double-blinded, parallel-groups trial, which was completed by 37 AUD patients. Randomization accounted for age, sex, and AUD severity. Randomization and blinding are described in greater detail in the original publication [24]. Note that a sample size of 60 participants was determined by power analysis, but could not be reached due to delays related to the COVID-19 pandemic [24]. Out of the 37 patients that completed the study, three refused to give blood samples. For additional three patients that dropped out before the last blood sampling (two from the placebo group, one from the psilocybin group), blood methylation data for the first two sampling time points was available. Non-completion of the study was associated with the drug consumption in all three cases (two patients relapsed on alcohol, one consumed cocaine after the second blood sampling).

Main inclusion criteria for the trial included an AUD diagnosis according to DSM-5 criteria, as well as detoxification from alcohol 6 weeks prior to enrolment in the study. Patients were excluded in case of major psychiatric comorbidities (schizophrenia, schizoaffective disorder, or psychosis) or a family history thereof, suicidality, substance use disorders other than alcohol and nicotine, or unstable medical conditions.

We included the blood samples from the drop-out participants in the EWAS described below, but not for the analyses that related methylation levels to psychometric data, as this data was not available for these patients. Within the 37 participants (female: 13, male: 24) that made up our sample, the average number of fulfilled DSM-V criteria was 7.51 (SD: 2.79), body mass index (BMI) 24.71 (SD: 3.66), and 21 were smokers. The age within the cohort ranged from 21 to 58 years (mean: 37.35; SD: 12.49).

**Fig. 1:**
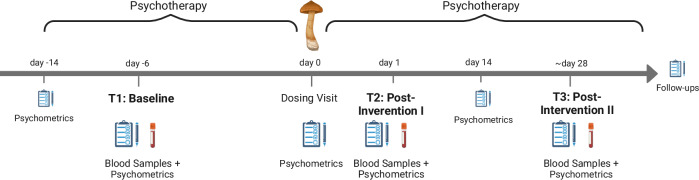
Overview of the clinical trial timeline (*n* = 37), conducted over six weeks. The study included three assessment days with blood sampling and acquisition of psychometrics: baseline (T1), 24 h post-intervention (T2), and ~28 days post-intervention (T3). Psilocybin (25 mg) or placebo (mannitol) was administered on day 0, the dosing visit, embedded within a broader psychotherapeutic framework incorporating preparatory and integrative sessions. Follow-up assessments were conducted after T3 to monitor longer-term outcomes.

The timeline of the trial (Fig. 1) included the acquisition of three blood samples: T1 (*n* = 37; baseline, two weeks before dosing visit), T2 (*n* = 37; one day after dosing visit), and T3 (*n* = 34; around four weeks after dosing visit). During the dosing visit, the treatment group (*n* = 18; after drop-out: *n* = 17) was administered 25 mg of psilocybin (acquired from Usona Institute, Madison, Wisconsin) orally while the placebo group (*n* = 19; after drop-out: *n* = 17) received an inactive placebo (mannitol). Blinding in clinical trials with psychedelics is challenging, given their subjective effects [27]. Mannitol, a sugar, was chosen as placebo because it does not produce subjective effects, allowing us to investigate the mechanisms of action of psilocybin.

Primary outcomes of interest were daily mean alcohol use in the four weeks after the dosing visit, as well as the time to relapse (≥1 standard unit of alcohol per day). Besides the primary outcomes, we included two secondary psychometric scores: Beck’s Depression Inventory (BDI) [28] and Beck Hopelessness Scale (BHS) [29]. For these variables, we calculated subject-wise Δ-values between T3 and T1 and used them for downstream analyses of the methylation data. More detailed information on study design, participants, inclusion and exclusion criteria, as well as psychometrics can be found in the original publication [24].

### Ethical approval

The clinical part of this study was approved by Swiss legal agencies (Cantonal Ethics Committee, Swiss Agency for Therapeutic Products [Swissmedic], Federal Office of Public Health [BAG]), and adhered to the revised declaration of Helsinki from 2000, as well as guidelines for Good Clinical Practice (GCP). Data sharing and processing adhered to the data protection laws outlined in the General Data Protection Regulation (GDPR) of the European Union. Patients gave informed consent to all experimental and data processing procedures.

### DNA extraction and DNA methylation assessment

DNA extraction and methylation analysis were carried out at Life&Brain GmbH in Bonn. 10 ml EDTA-treated whole blood samples were used for DNA extraction via Chemagen Chemagic Systems technology. Extracted DNA was screened for genotypic variants on Illumina’s Infinium Global Screening Array-24 (GSA) v3.0 to include single-nucleotide polymorphism (SNP) outlier analysis in the pre-processing of methylation data. After bisulfite conversion of the DNA, CpG methylation was assessed on Illumina’s Infinium MethylationEPIC BeadChip v2.0, yielding raw data with unmethylated and methylated signal intensities for each of the ~ 950,000 probes stored in idat files, which were then processed as described below.

### Statistical analysis

All statistical analyses were performed in the *R* statistical environment (version 4.2.1 and 4.3.0; https://www.r-project.org/).

### Data preprocessing

Preprocessing was based on the CPACOR pipeline [30] and included filtering for sample call rate, sex mismatches, genetic outliers, and cross-reactive probes, as described previously [31]. For estimation of genetic outliers, genotype data were preprocessed as previously described [32], reduced to 20 dimensions by principal component analysis (PCA), and samples for which a component’s loading coefficient differed by 4.5 standard deviations from the mean would have been removed. No such outliers were found. We estimated cell type composition [33, 34] and performed PCA on cell type data and internal control probes to extract covariates for statistical modeling. Duplicated CpG sites on the EPIC array were excluded at random. Eventually, 817 247 CpG sites were included in statistical analyses.

### Mixed linear model on individual CpG sites

Using the R package lme4 [35], we calculated mixed linear models on the M-values [36], with group (psilocybin vs. placebo) as a between and time as a within factor. We included a random effect for patient ID to account for inter-subject variability, one cell type PC and two control probe PCs as covariates, as well as sex, age, smoking, and daily alcohol intake before withdrawal (in units per day). For variance decomposition [37] and multicollinearity analysis, see Sup. Fig. 1 and 2, respectively. With its small size (*n* = 37), this study is statistically underpowered to reach conservative EWAS thresholds (α ≈ 10⁻^6^ – 10⁻⁸) [38, 39]. Therefore, we report genome-wide significance at a suggestive threshold of *p* = 1e-5, as commonly done in such scenarios [21, 40–42]. For the sake of completeness, *p*-values corrected for false discovery rate (FDR) are given in the Supplementary Tables. M-values were used to improve normality and variance stability relative to β-values [36], which is advantageous for linear modeling. Diagnostic plots (QQ-plots and residuals vs fitted values) confirmed normality and homoscedasticity for model fits with longitudinal *p* < 0.001 and |Δβ| > 0.02.

Primary effects of interest in the linear model were the interaction effects time2*group and time3*group, indicating significant differences in change from baseline methylation between treatment and placebo groups, 24 h and 28 days after the intervention, respectively. Significant longitudinal effects were *post hoc* tested by running cross-sectional *t*-tests for the relevant time points (T2 or T3, respectively) on the beta values adjusted for the covariates (using *R*’s predict function). We report goodness-of-fit for models with significant effects as variance explained by all regressors in the model [43] (conditional R^2^ calculated with performance library [44]). Model estimates resulting in singular fits were excluded from subsequent analyses, leaving 649 975 CpG sites in the dataset. Results were annotated using the manufacturer’s manifest (https://emea.support.illumina.com/array/array_kits/infinium-methylationepic-beadchip-kit/downloads.html). Non-annotated CpGs that appeared as relevant in the *post hoc* tests, *i.e*., significant effects with a magnitude of |Δβ| > 0.02 for the cross-sectional difference, were also screened on https://ewascatalog.org/ for associated genes.

### Sensitivity analysis

A sensitivity analysis using G*Power 3.1 [45] to estimate the effect size required to detect a deviation from zero of the total explained variance R^2^ in a linear multiple regression model was calculated. Parameters used here were α = 1e-5, *n* = 37, Power = 0.8, and number of predictors = 9.

### Downstream analyses

**Fig. 2:**
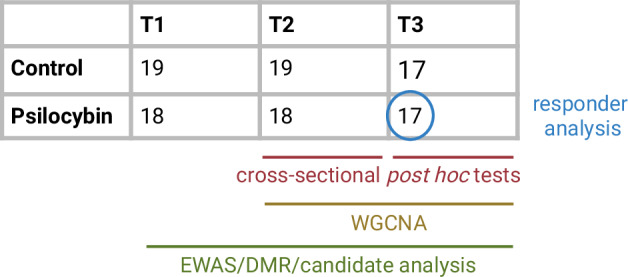
Sample sizes per group and time point, and inclusion in different analysis streams. Longitudinal linear modeling of the EWAS incorporated all time points, leading to *n*_*samples*_ = 108. DMR and candidate analysis are based on EWAS results. Cross-sectional *post hoc* tests were performed for T2 (*n*_*samples*_ = 37) and T3 (*n*_*samples*_ = 34). The WGCNA included only T2 and T3 (*n*_*samples*_ = 71) to focus the analysis on potential treatment effects. The responder analysis was conducted on participants of the psilocybin groups that completed the study, leading to *n*_*samples*_ = 17 at T3, although the samples were taken from baseline T1.

Sample sizes for the different downstream analysis are detailed in Fig. 2. Differentially methylated regions (DMR) were detected using the dmrff algorithm [46] (maximal gap size: 1 000 bp; *p*-cutoff: 0.05). Visualization was based on the qqman package [47] and used the *p*-values for longitudinal effects. We considered DMRs with a minimum coverage of two CpG sites and report the results of cross-sectional *post hoc* tests for the affected CpGs.

Weighted Correlation Network Analysis (WGCNA) [48] was performed on the 5% most variable CpG sites (45 962 CpGs) from the two post-intervention timepoints to derive co-methylation modules that capture potential treatment effects. Networks were constructed using the following parameters: soft power threshold = 3 (defined by the criterion of approximate scale-free topology: truncated R^2^ > 0.90), minimum module size = 50, mergeC Figure 2 utHeight = 0.25, and maxBlockSize = 46 000. In WGCNA, modules are labeled by colors. The module’s eigen-CpGs (analogous to eigengenes [48]), representing a weighted average of the module’s expression profile, were calculated and correlated with phenotypic variables of interest: group, duration of abstinence, and mean alcohol intake during the 4-week post-treatment period, as well as Δ-values (T3-T1) for BDI and BHS. Eigen-CpG methylation values were winsorized to two standard deviations. For each variable, we report the module with the strongest correlation, including the relation between module membership (correlation between methylation of CpG site and module’s eigen-CpG) and CpG significance (-log(*p*) of correlation between CpG methylation and trait of interest) [48].

Furthermore, we performed Gene Ontology Overrepresentation Analysis (GO ORA) using missMethyl [49]. This was done on the CpG sites included in the co-methylation modules we report.

### Candidate gene analysis

We also examined methylation changes at CpG sites in a selection of candidate genes chosen based on their proposed involvement in AUD and/or the effects of psychedelics. This selection comprised (i) receptors presumably involved in the effects of or targeted by psilocin, the active metabolite of psilocybin [50], namely *HTR2A, HTR1A, SLC6A4, NTRK2, GRM2, DRD1*, and *DRD2* [51–56]; (ii) immediate early genes (IEGs) and plasticity-related genes associated with addictive disorders and/or psychedelic drug action: *EGR1, EGR2, FOSB, JUND, BDNF* and *SV2A* [56–64]*;* and a more heterogeneous group (iii) consisting of genes related to inflammation (*TNF, IL6, CXCL8)*, glutamate (*GRIN2B*) and glucocorticoid (*NR3C1*) signaling, as well as epigenetic regulation (*HDAC2*) that are implied to play a role in AUD [65–68].

330 CpG sites annotated to candidate genes were retrieved and screened for nominally significant longitudinal effects (*p* < 0.05). Significant CpGs were *post hoc* tested for cross-sectional differences at T2 or T3, respectively, using *t*-tests on β-values adjusted for the covariates from the linear model. We report CpGs with significant (*p* < 0.05) cross-sectional differences that exceeded |Δβ| = 0.02.

Furthermore, we screened for baseline differences in the candidate CpGs between treatment responders (<1 standard unit of alcohol during 4-week follow-up; *n* = 6) and non-responders (≥1 standard unit of alcohol during follow-up; *n* = 11). Due to the small sample sizes for this comparison (*n* = 17), non-parametric Wilcoxon tests were chosen, and results for *p* < 0.05 uncorrected are reported.

### Mediation analysis

We conducted a mediation analysis to explore whether changes in methylation mediated the effects of psilocybin treatment on depression scores (ΔBDI/ΔBHS), focusing on eight CpG sites with prior significance. These CpGs sites were the ones with cross-sectional differences of |Δβ| > 0.02, identified in the EWAS, the DMR, and the candidate analysis. Methylation changes (ΔT2-T1 or ΔT3-T1) were modeled by group (mediator model), and ΔBDI/ΔBHS was modeled by methylation and group (outcome model). Methylation values were adjusted using prior mixed model predictions. As methylation showed no significant effect in the outcome models, mediation analysis was not pursued further.

## Results

### Clinical outcomes

A detailed description of the clinical results can be found in Rieser et al. [24]. Drinking-related outcome metrics did not show significant differences: duration of abstinence had a mean of 11.24 days after placebo versus 17.77 days after psilocybin (*t* = −1.7, df = 31.6, *p* = 0.095, Cohen’s d = −0.59); mean daily alcohol intake was 1.39 units after placebo and 0.84 units after psilocybin (*t* = 1, df = 26.9, *p* = 0.331, Cohen’s d = 0.34). We also analyzed group differences in ΔBDI and ΔBHS (Δ: T3-T1) and found significant effects: ΔBDI = −0.41 in the placebo group vs. ΔBDI = −6.18 after psilocybin (*t* = 2.5, df = 31.2, *p* = 0.017, Cohen’s d = 0.87), as well as ΔBHS = 0.65 in the placebo group and ΔBHS = −1.59 after psilocybin for ΔBHS (*t* = 2.5, df = 31.9, *p* = 0.017, Cohen’s d = 0.86).

### EWAS results

**Fig. 3:**
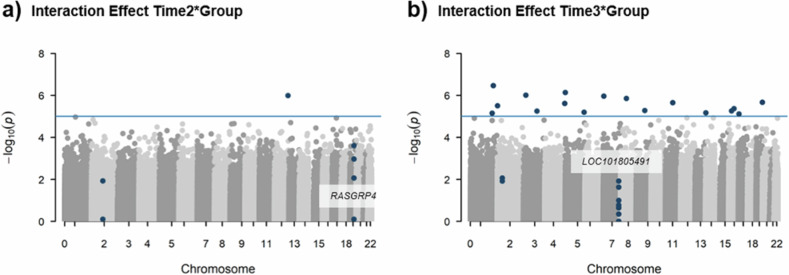
Results of linear modeling and DMR analysis. **a** Manhattan plot showing the -log(*p*)-values for the longitudinal effects time2*group at each CpG against its location in the genome. Blue line indicates genome-wide significance cutoff of *p* < 1e-5. Blue dots indicate CpGs that reach genome-wide significance or belong to a DMR. Where possible, DMRs are annotated to genes. **b** Same as a) but for longitudinal effects time3*group.

Linear modeling identified one intergenic CpG site with a significant interaction effect (*p* < 1e-5; Fig. 3a & Sup. Tab. 1.1) between group and time point 2; cg04492946 (*t*_*LMM*_ = 5.4, df_*LMM*_ = 64.5, *p* = 1e-6, R^2^ = 0.72). However, *post hoc* testing revealed no significant cross-sectional difference (*t*_*post*_ = −0.4, df_*post*_ = 35, *p* = 0.714, Δβ = 0.003). At T3, 17 CpG sites showed significant longitudinal effects (*p* < 1e-5; Fig. 3b & Sup. Tab. 1.2). *Post hoc* analysis of cross-sectional group differences identified four CpGs with an effect size of |Δβ| > 0.02 (negative Δβ indicates lower methylation in psilocybin group): cg01405499 (*t*_*post*_ = 2.5, df_*post*_ = 32, *p* = 0.017; Δβ = −0.02; R^2^ = 0.74), cg23107740 (*t*_*post*_ = 6.8, df_*post*_ = 32, *p* = 1.1e-7; Δβ = −0.04; R^2^ = 0.48), cg09767929 (*t*_*post*_ = −9.4, df_*post*_ = 32, *p* = 9.9e-11; Δβ = 0.03; R^2^ = 0.38), and cg17174681 (*t*_*post*_ = −1.1, df_*post*_ = 32, *p* = 2.1e-12; Δβ = 0.02; R^2^ = 0.43). None of these CpG sites were annotated to genes in the Illumina manifest. Using https://ewascatalog.org/, however, cg23107740 was annotated to transducin-like enhancer of split 4 (*TLE4)* and cg01405499 to the non-coding RNA *LINC01250*. Of note, cg23107740 displayed a significant group difference at baseline, which was inverted at time point 3 (*t* = −4.4, df = 35, *p* = 1.1e-4, Δβ = 0.02; R^2^ = 0.48; Sup. Fig. 3).

### Sensitivity analysis

Sensitivity analysis revealed a minimal effect size of *f*^*2*^ = 2.34 for our models. Based on ${R}^{2}=\,{f}^{2}/(1+{f}^{2})$, a model fit needs to explain a proportion of at least R^2^ = 0.7 in the methylation values to produce accurate findings with a power of 0.8 in our study.

### Differentially methylated regions (DMR)

Two DMRs showed psilocybin-dependent longitudinal effects at T2 and T3, respectively. DMRs are highlighted in Fig. 3a and b. One DMR associated with psilocybin-dependent changes at T2 was intergenic (*n* = 2 CpGs; z = 5.49; *p*_*FDR*_ = 0.026), the other one covered CpGs in the gene RAS (rat sarcoma) guanyl nucleotide-releasing protein 4 (*RASGRP4*) (*n* = 4 CpGs; z = 6.23; *p*_*FDR*_ = 3.2e-4). Longitudinal psilocybin-dependent effects at T3 also covered two regions: one intergenic DMR (*n* = 7 CpGs; z = −6.47; *p*_*FDR*_ = 6.6e-5) and one in the non-coding RNA *LOC101805491* (*n* = 2 CpGs; z = −6.08; *p*_*FDR*_ = 8e-4; annotated using https://ewascatalog.org/). Cross-sectional *post hoc* tests of the covered CpGs revealed that only one affected methylation site displayed a significant cross-sectional effect of |Δβ| > 0.02. This was cg14565721 in *RASGRP4* gene showing hypermethylation 24 h after psilocybin (*t* = −2.1, df = 35, *p* = 0.041; Δβ = 0.02; R^2^ = 0.88). The four CpG sites in this DMR lie within a 2 000 bp distance from the transcription start site of *RASGRP4*, suggesting potential involvement in transcription regulation.

### Weighted correlation network analysis (WGCNA)

**Fig. 4:**
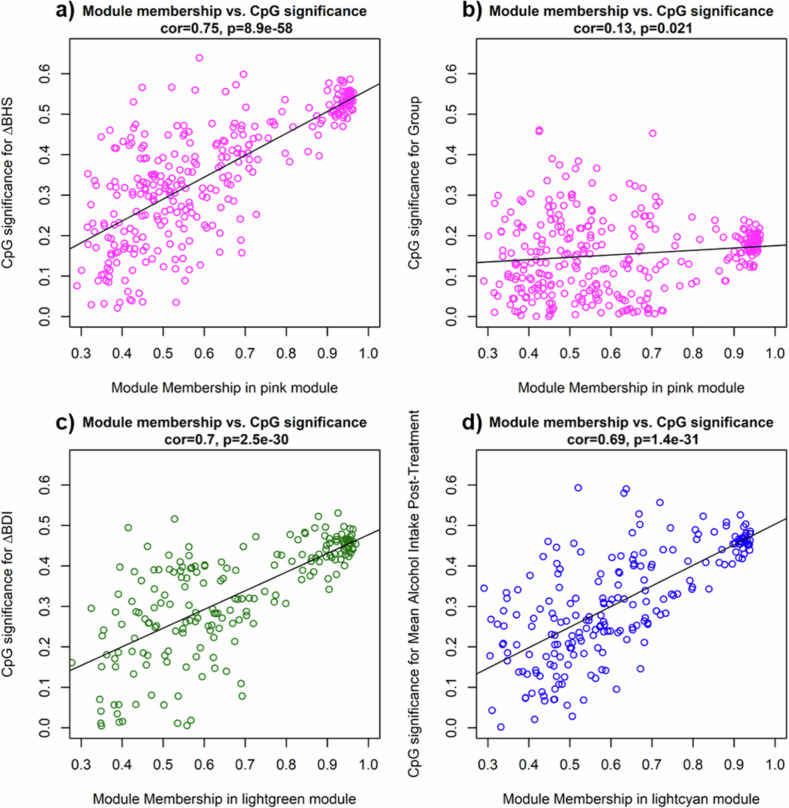
Co-methylation modules with significant correlation between CpG significance for variable of interest and module membership. Colors indicate the co-methylation module. The regression line indicates the strength of the association. **a** Membership pink module vs. CpG significance ΔBHS. **b** Membership pink module vs. CpG significance group. **c** Membership lightgreen module vs. CpG significance ΔBDI. **d** Membership lightcyan module vs. CpG significance mean alcohol intake during 4-week follow-up.

WGCNA identified 34 co-methylation modules (median size: *n* = 206; range: *n* = 73-17612). Significant correlations between module eigengenes and the variables of interest occurred in the following modules: pink (group: *r* = −0.24, df = 33, *p* = 0.044; ΔBHS: *r* = 0.56, df = 33, *p* = 5.2e-7), lightgreen (ΔBDI: *r* = 0.48, df = 33, *p* = 2e-5), lightcyan (mean alcohol use: *r* = 0.47, df = 33, *p* = 3.5e-5), green (duration of abstinence: *r* = 0.27, df = 33, *p* = 0.021). For most modules, the relationship between modules and the respective variables was confirmed by strong correlations between module membership and gene significance for the CpG sites within the modules [48] (Fig. 4): pink (ΔBHS: *r* = 0.75, df = 311, *p* = 8.9e-58), lightgreen (ΔBDI: *r* = 0.7, df = 195, *p* = 2.5e-30), lightcyan (mean alcohol use: *r* = 0.69, df = 212, *p* = 1.4e-31). The relationship between the pink module pink and group assignment was weak (*r* = 0.13, df = 311, *p* = 0.021) and only for the green module was this correlation insignificant (duration of abstinence: *r* = 0.06, df = 465, *p* = 0.2).

### Gene ontology overrepresentation analysis (GO ORA)

GO term analyses on the co-methylation modules revealed enrichment of terms broadly related to neurodevelopment and (lightgreen module, Sup. Tab. 2.1), immune function and cell cycle regulation (pink module, Sup. Tab. 2.2), synaptic transmission and intracellular protein regulation (lightcyan module, Sup. Tab. 2.3), as well as calcium signaling and gene/protein regulation (green module, Sup. Tab. 2.4), among other functions. However, no enrichment survived correction for false discovery rate (FDR).

### Candidate analysis

**Fig. 5:**
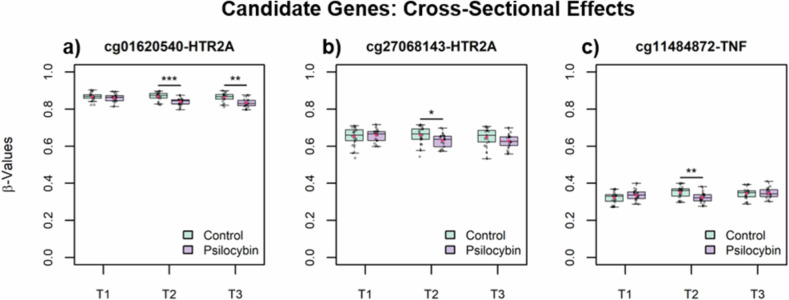
CpG sites that showed significant cross-sectional effects in *post-hoc* testing of candidate genes. Green is the placebo group, violet is the psilocybin group. *: *p* < 0.05; **: *p* < 0.01; ***: *p* < 0.001. **a** cg01620540 in the *HTR2A* promoter. **b** cg27068143 in the *HTR2A* promoter. **c** cg11484872 in the *TNF* promoter.

Among the 330 target CpG sites investigated, 19 reached nominal significance (*p* < 0.05) for the interaction effect of time and group at T2 and 16 for the interaction effect at T3 (see Sup. Tab. 3.1 & 3.2). None of these effects remained significant after FDR correction. Among the significant CpGs, three sites showed significant cross-sectional effects exceeding |Δβ| = 0.02 (see Fig. 5): cg01620540 (T2: *t* = 3.8, df = 35, *p* = 4.8e-4, Δβ = −0.03; T3: *t* = 3.4, df = 32, *p* = 1.6e-3, Δβ = −0.03, R^2^ = 0.56) and cg27068143 (T2: *t* = 2, df = 35, *p* = 0.049, Δβ = −0.03, R^2^ = 0.75), both of which lie within 2 000 bp up/downstream of the transcription start site of *HTR2A*, as well as cg11484872 (T2: *t* = 2.9, df = 35, *p* = 7e-3, Δβ = −0.03, R^2^ = 0.54) which is associated with the *TNF* promoter. Again, promoter association/proximity to the transcription start site of the CpG sites implies potential regulatory functions.

Next, we compared methylation levels of the candidate CpGs between responders (abstinent until 4-week follow-up) and non-responders in the psilocybin group. This comparison needs to be pointed out as highly exploratory given the small sample size of *n* = 17. We identified 12 CpGs that displayed nominally significant differences before the psilocybin treatment (Table 1).

**Table 1:** Significant (*p* < 0.05) baseline differences between treatment responders and non-responders.

CpG	*p*	Δβ	FDR	Gene	EntrezID	Location
cg12067298	0.037	−0.028	0.988	BDNF	627	TSS1500
cg15710245	0.007	0.03	0.8	BDNF	627	TSS1500
cg11501905	0.027	0.069	0.988	DRD1	1812	exon
cg19730798	0.048	−0.005	0.988	DRD2	1813	TSS200
cg00105415	0.015	0.076	0.8	EGR2	1959	TSS1500
cg02881684	0.048	−0.072	0.988	FOSB	2354	exon
cg24609211	0.027	−0.052	0.988	FOSB	2354	exon
cg15287403	0.015	0.007	0.8	GRM2	2912	exon
cg00221070	0.037	−0.014	0.988	HTR1A	3350	exon
cg08652028	0.01	0.049	0.8	NR3C1	2908	exon
cg13848734	0.007	0.01	0.8	NTRK2	4915	TSS1500
cg14312898	0.015	0.011	0.8	SLC6A4	6532	TSS1500

*p-*values stem from Wilcoxon-tests. Δβ**-**values represent the contrast responders > non-responders. FDR column shows multiple comparison corrected *p*-values. Gene annotation derived from the manufacturer’s manifest. Location describes the position of the CpG sites within the gene: TSS = (distance from) transcription start site; i.e., TSS1500 describes a CpG site within 1 500 bp distance from that transcription start site, which suggests potential relevance for the regulation of transcription.

### Mediation analysis

Upon testing the eight statistically and biologically relevant CpG sites identified in our analyses on psilocybin treatment, we observed no significant effects for methylation in the outcome models for either ΔBDI or ΔBHS, rendering mediation analysis obsolete.

## Discussion

We present the first methylome-wide exploration of psilocybin-induced changes in blood DNA methylation in a clinical population (*n* = 37). In this analysis, we identified a number of CpG sites and co-methylation modules with potential relevance for psilocybin’s effects that may support future hypothesis-driven research.

In our EWAS, four CpG sites showed significant methylation changes after psilocybin. One of these was annotated to a gene, *TLE4*, where it is located in the gene body [69]. *TLE4* is a transcriptional co-regulator involved in developmental [70–72] and immunoregulatory [73, 74] processes. Furthermore, *TLE4* regulates maturation and maintenance of corticothalamic projection neuron identity [72], Schwann cell differentiation [75], and post-synaptic gene transcription at neuromuscular junctions [76]. *TLE4* has also been implicated in addictive behavior in a preclinical study on oxycodone self-administration [77]. Given this context and the suggested role of structural plasticity in psilocybin’s therapeutic effects [78], there may be a relationship between psilocybin-induced alterations in *TLE4* methylation and potential neuroplastic effects of psilocybin in AUD.

Furthermore, we discovered four DMRs associated with psilocybin-induced methylation changes. One DMR implicated in effects at T2 covered a gene, *RASGRP4*. This signaling molecule contributes to the development of mast cells [79] as well as the regulation of immune responses [80, 81]. Psychedelics, including psilocybin, possess immunomodulatory capacities [82, 83], and reductions in neuroinflammation may contribute to their lasting psychological benefits [84]. In AUD, on the other hand, (neuro)inflammatory processes are upregulated [85], seemingly exacerbating cognitive symptoms associated with this condition [86]. Methylation changes in *RASGRP4* may reflect, at least in part, psilocybin’s immunomodulatory effects. While the *RASGRP4* methylation change, as well as the *TLE4* effect, represent psilocybin-associated effects co-occurring with reduced depression symptoms at the group level, a direct mediating role is unlikely for both, as indicated by the negative mediation analysis.

WGCNA revealed several co-methylation modules associated with either treatment group or the drinking-/depression-related psychometrics, making the distinction of effect classes especially important in this analysis. Only the pink module showed significant correlation with the treatment, as well as with one of the psychometric measures (ΔBDI), suggesting that methylation of the involved loci might fulfill a mediating role in psilocybin-induced relief of depressive symptoms. The involvement of genes relevant to immune function and cell cycle regulation in this module, again, indicates a relationship between the potential effects of psilocybin on the immune system and its anti-depressive capacities, as implied by previous research [84]. Interestingly, neuroinflammation has been suggested as a link between AUD and major depression before [87] and might represent a common target for psilocybin’s effects across these disorders. The other modules correlating with ΔBHS, duration of abstinence, and mean alcohol use during the 4-week follow-up, on the other hand, did not relate to the psilocybin treatment and seem to fall in the category of effects related to abstinence or reduced symptom load. It is known that DNA methylation patterns in AUD change during prolonged abstinence [88, 89], for instance, involving gene loci related to immune function [90] and neuroplasticity [91]. As the modules covered biological processes related to synaptic transmission and gene transcription, they possibly describe such abstinence-related methylation changes that are independent of psilocybin treatment.

The candidate gene analysis revealed evidence for psilocybin-induced hypomethylation in two CpG sites within the promoter of *HTR2A*, which codes for the primary molecular target of psychedelics, the 5HT2a receptor. Aberrant methylation of *HTR2A* has been associated with psychiatric symptoms such as impulsivity in cocaine use disorder [92], or depressive rumination in people suffering from adverse childhood experiences [93, 94]. Normalization of such *HTR2A* methylation might thus lead to symptom relief in patients suffering from conditions like depression or AUD. We also observed a transient hypomethylation in a CpG site in the *TNF* promoter after psilocybin. This may represent a temporary influence on immune signaling. Interestingly, psilocybin decreases *TNF* blood levels in the short term [84]. As with the EWAS and DMR results, these drug-induced effects accompany reductions in depressive symptoms at the group level, without clear evidence for a mediating role.

Lastly, the descriptive examination of baseline differences between responders to psilocybin treatment (abstinent at 4-week follow-up) and non-responders revealed nominally significant differences in several CpG sites related to neuronal plasticity (*BDNF*, *NTRK2*, *EGR2*, *FOSB*) and various neurotransmitter systems (*DRD1*, *DRD2*, *GRM1*, *HTR1A*, *SLC6A4*, *NR3C1*). The search for predictors of psychedelic treatment responsivity is ongoing and currently focuses on phenomena like the acute effects of psilocybin on brain activity and phenomenology, or changes in language patterns shortly after substance intervention [95, 96]. Less is known about molecular factors that could predict treatment responses before psychedelics are administered, and the genes identified here might provide a starting point for future research focusing on biomarkers of treatment responsivity.

### Limitations

This study is subject to some limitations. Firstly, as the primary endpoints of the RCT were not significantly improved [24], the present dataset cannot provide a definitive biomarker for AUD treatment. Nonetheless, the reductions in depressive symptoms – which frequently co-occur with AUD [25, 26] – together with a lack of previous methylome-wide screenings of psychedelic effects in clinical populations, justify this exploratory investigation. Secondly, the results presented here need to be seen as hypothesis-generating, since the sample size was modest and the analyses lacked sufficient statistical power to satisfy conservative α-thresholds commonly used in EWAS [38, 39]. Accordingly, most findings did not survive multiple comparison corrections, and most model fits remain below our R^2^ threshold for sufficient power. Furthermore, on average, the effect sizes we observed are small. Consequently, a single administration of psilocybin appears unlikely to cause strong and persistent effects on DNA methylation. The power issues here affect the initial linear modeling as well as downstream analyses like the WGCNA or GO ORA and the investigation of baseline differences. Lastly, our methylome analysis is based on blood samples instead of neuronal tissue. Given the variability of methylomic signatures between tissue types [97], mechanistically interpreting the effects of psychopharmaceuticals based on blood methylome data remains speculative. A blood-brain concordance analysis [97] of the significant results from our candidate analysis illustrates this issue: correlations for the same CpG site differed between cortical regions, while within a single brain region CpG sites from the same promoter region showed inconsistent patterns (Sup. Fig. 5). Despite this caveat, blood remains the most pragmatic tissue for clinical biomarker discovery which lends blood-based EWAS a high translational value.

## Conclusion

This exploratory analysis presents potential novel epigenetic associations with psilocybin treatment for AUD patients, indicators for methylation changes in genes involved in serotonin and immune signaling, as well as possible methylomic predictors of treatment responsivity. Future RCTs on psilocybin should incorporate molecular endpoints to enable cross-study data integration with the prospect of identifying reliable biomarkers for clinical usage. If replicated, our findings suggest that immunomodulatory mechanisms contribute to psilocybin’s anti-depressive – and possibly anti-addictive – effects, thereby presenting a potential therapeutic avenue for comorbid populations. Apart from that, further research to confirm our findings could comprise probing the methylomic effects of psilocybin in blood and neuronal cell cultures derived from AUD patients or in brain tissue from rodent models of AUD.

## Supplementary information

Supplementary Material

Supplementary Figure 1

Supplementary Figure 2

Supplementary Figure 3

Supplementary Figure 4

Supplementary Figure 5

Supplementary Tables

## Data Availability

Data will be made available on the European Genome Archive (EGA).

## Code availability

Code is available upon request.
