# Supplementary material for: Epigenome-wide association study of psilocybin-induced methylome changes in alcohol use disorder
Source: Transl Psychiatry. 2026 May 26;16:283. doi: 10.1038/s41398-026-03961-3 (PMC13212986; doi:10.1038/s41398-026-03961-3)
Supplement: Supplementary file 1 — Supplementary Material [file 41398_2026_3961_MOESM1_ESM.docx]

# Supplementary Material

**Sup. Fig. 1**: Variance decomposition with covariates included in linear model. PC1: cell type principal component 1; PC1/2_cp: control probe principal component 1/2.

**Sup. Fig. 2**: Pearson correlation matrix with regressors included in the model. PC1: cell type principal component 1; PC1/2_cp: control probe principal component 1/2.

**Sup. Fig. 3**: Cross-sectional effects of cg23107740 methylation. Already at baseline, there was a random group difference that inverted throughout the experimental procedure.

**Sup. Fig. 4**: Pearson correlation matrix for com-methylation module eigen-CpGs and phenotypic traits. Upper value in each cell is Pearson’s *r*, lower value is *p*. Delta_BDI: change in Beck’s Depression Inventory between T1 and T3; Delta_BHS: change in Beck Hopelessness Scale between T1 and T3.

**BECon Screening**

To allow for potential mechanistic interpretations of our findings, we made use of the Blood–Brain Epigenetic Concordance (BECon; https://redgar598.shinyapps.io/BECon/) tool to examine CpG sites that emerged as relevant in the previous analyses (i.e., cross-sectional differences of |Δβ| > 0.02, identified in the EWAS, the DMR, and the candidate analysis). BECon is a web application that, based on an existing dataset from 16 subjects, provides information about the correlation of DNA methylation in blood and different brain areas. The brain areas included in this data set include Brodmann areas (BAs) 7, 10, and 20, corresponding to the precuneus/superior parietal lobule, frontopolar prefrontal cortex, and a part of the temporal cortex, respectively. BECon reports the Spearman correlation between blood and the different BAs, the variability of CpG methylation in each tissue type, as well as the dependence of the variability on cell type composition for brain and blood. Note that higher variability of a CpG site’s methylation is a desired trait for using it in EWAS as stable methylation of a locus diminishes its informative value in trait associations.

**Sup. Fig. 5**: Results of BECon screening

Information on blood brain correlations were available for three out of the eight CpG sites, namely the three CpGs identified in the candidate gene analysis (cg01620540, cg27068143, cg11484872).

The results of the blood-brain concordance screening with BECon are summarized in Sup. Fig. 5 and suggest a potential positive correlation of low to moderate size (*r* = 0.26) between methylation of cg01620540 in blood samples and the frontopolar prefrontal cortex (BA10), as well as moderate (*r* = -0.3) and strong (*r* = -0.56) negative associations with BA20 in the temporal cortex and BA7 (precuneus/superior parietal lobule), respectively. For cg11484872, strong (*r* = -0.51) and moderate (*r* = -0.37) negative correlation between blood and BA20/BA7, respectively, were identified.

The inconclusive nature of the observed correlations illustrates the difficulties that arise when mechanistically interpreting psilocybin effects using blood methylation. It needs to be highlighted that the participants in the BECon databank did not suffer from alcohol use disorder and the dataset used to create the tool was relatively small for an omics resource (*n* = 16), thus the results show in Sup. Fig. 5 might not directly translate to our data but rather serve a demonstrative purpose.
