# Supplementary figures and images for: Epigenome-wide association study of psilocybin-induced methylome changes in alcohol use disorder
Source: Transl Psychiatry. 2026 May 26;16:283. doi: 10.1038/s41398-026-03961-3 (PMC13212986; doi:10.1038/s41398-026-03961-3)

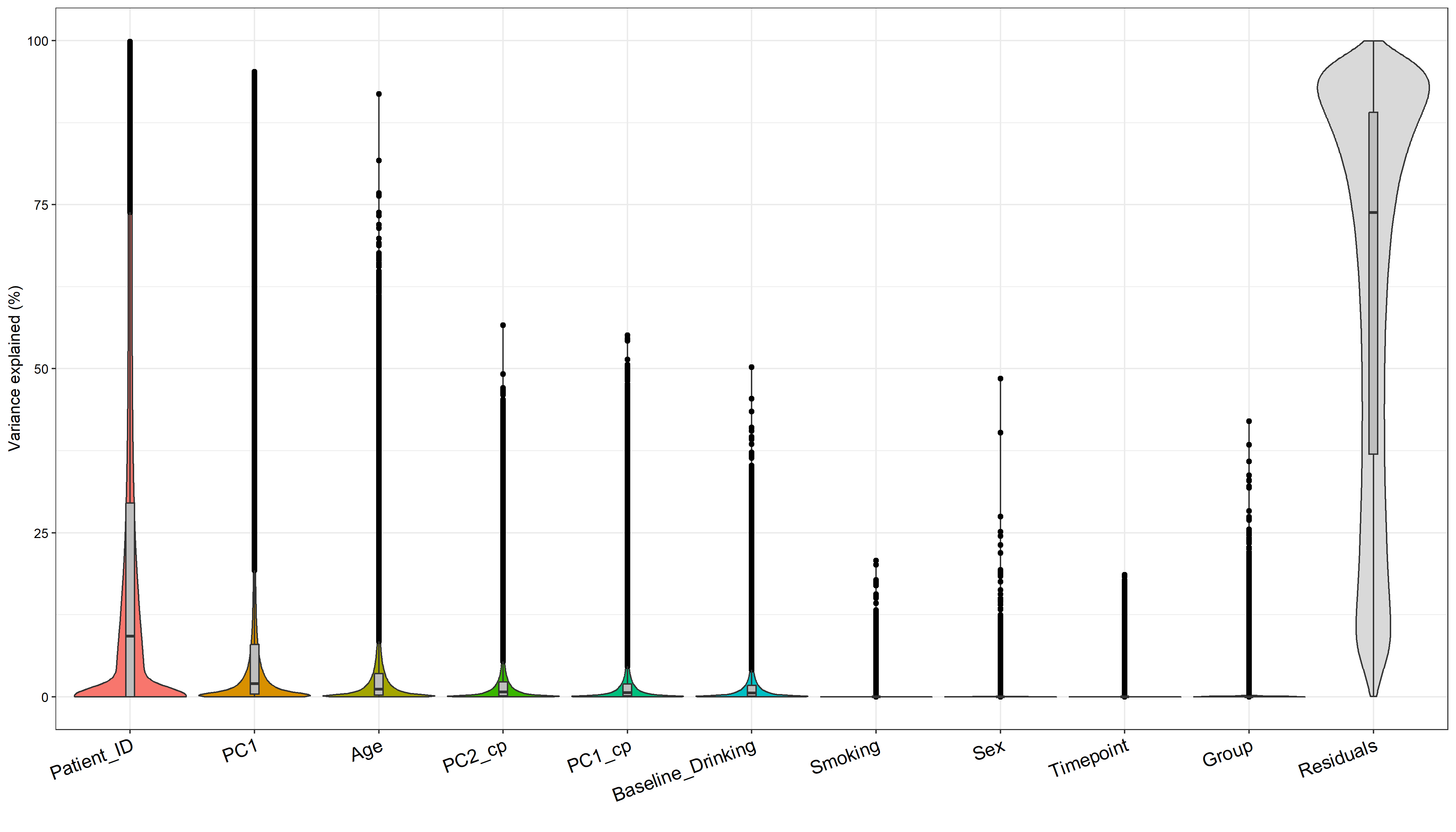

Supplement: Supplementary file 2 — Supplementary Figure 1 [file 41398_2026_3961_MOESM2_ESM.png]

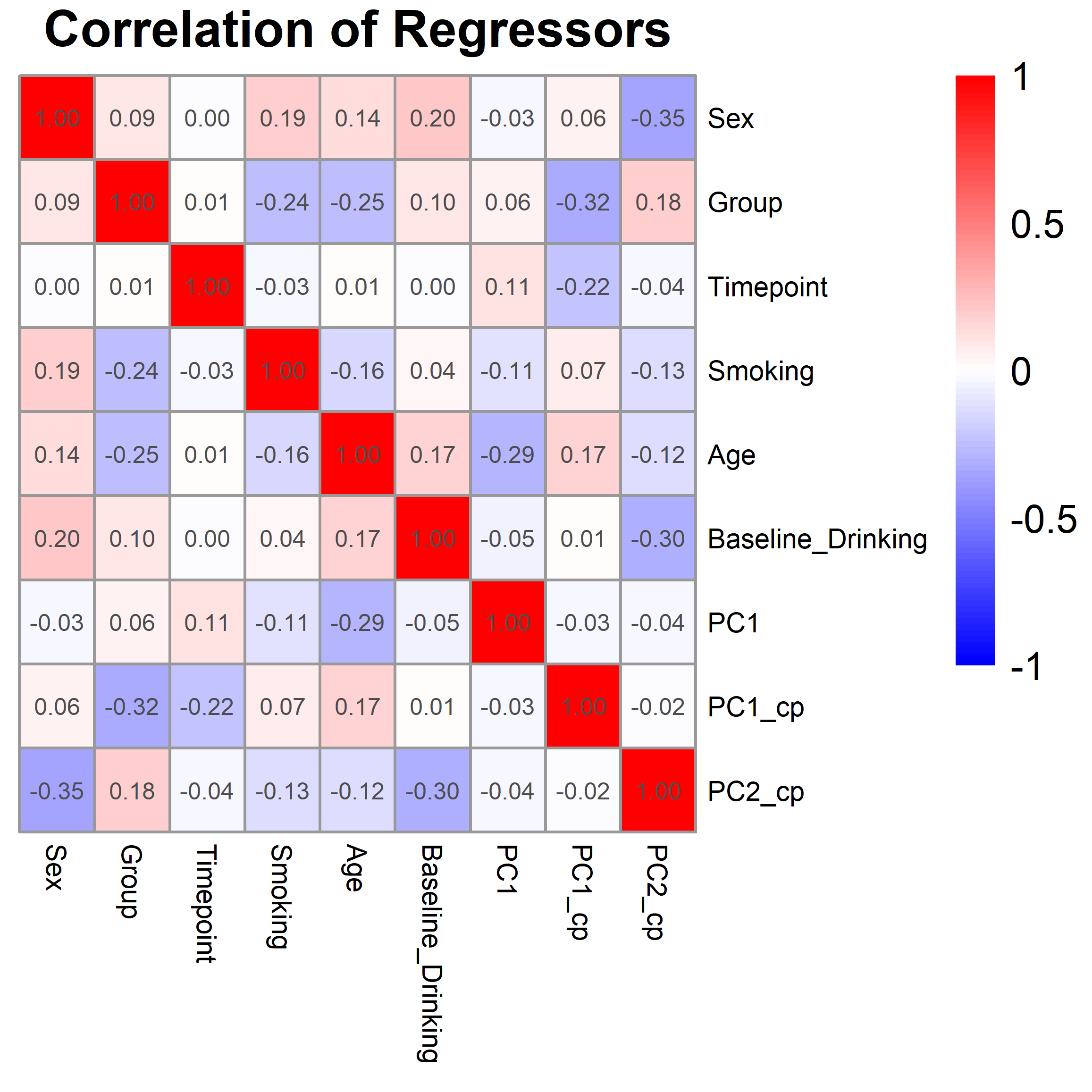

Supplement: Supplementary file 3 — Supplementary Figure 2 [file 41398_2026_3961_MOESM3_ESM.png]

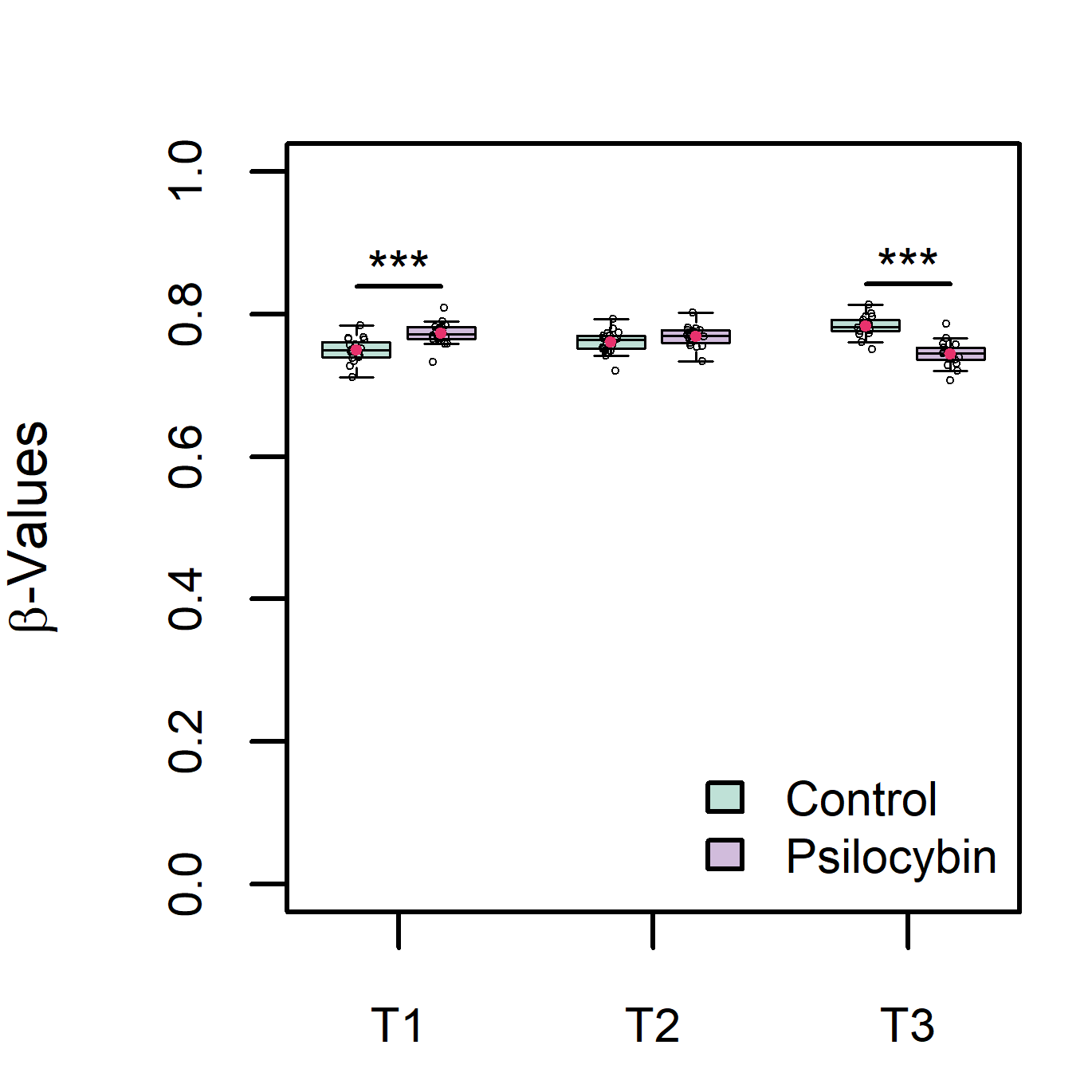

Supplement: Supplementary file 4 — Supplementary Figure 3 [file 41398_2026_3961_MOESM4_ESM.png]
