# Supplementary material for: Epigenome-wide association study of psilocybin-induced methylome changes in alcohol use disorder
Source: Transl Psychiatry. 2026 May 26;16:283. doi: 10.1038/s41398-026-03961-3 (PMC13212986; doi:10.1038/s41398-026-03961-3)
Supplement: Supplementary file 5 — Supplementary Figure 4 [file 41398_2026_3961_MOESM5_ESM.pdf]

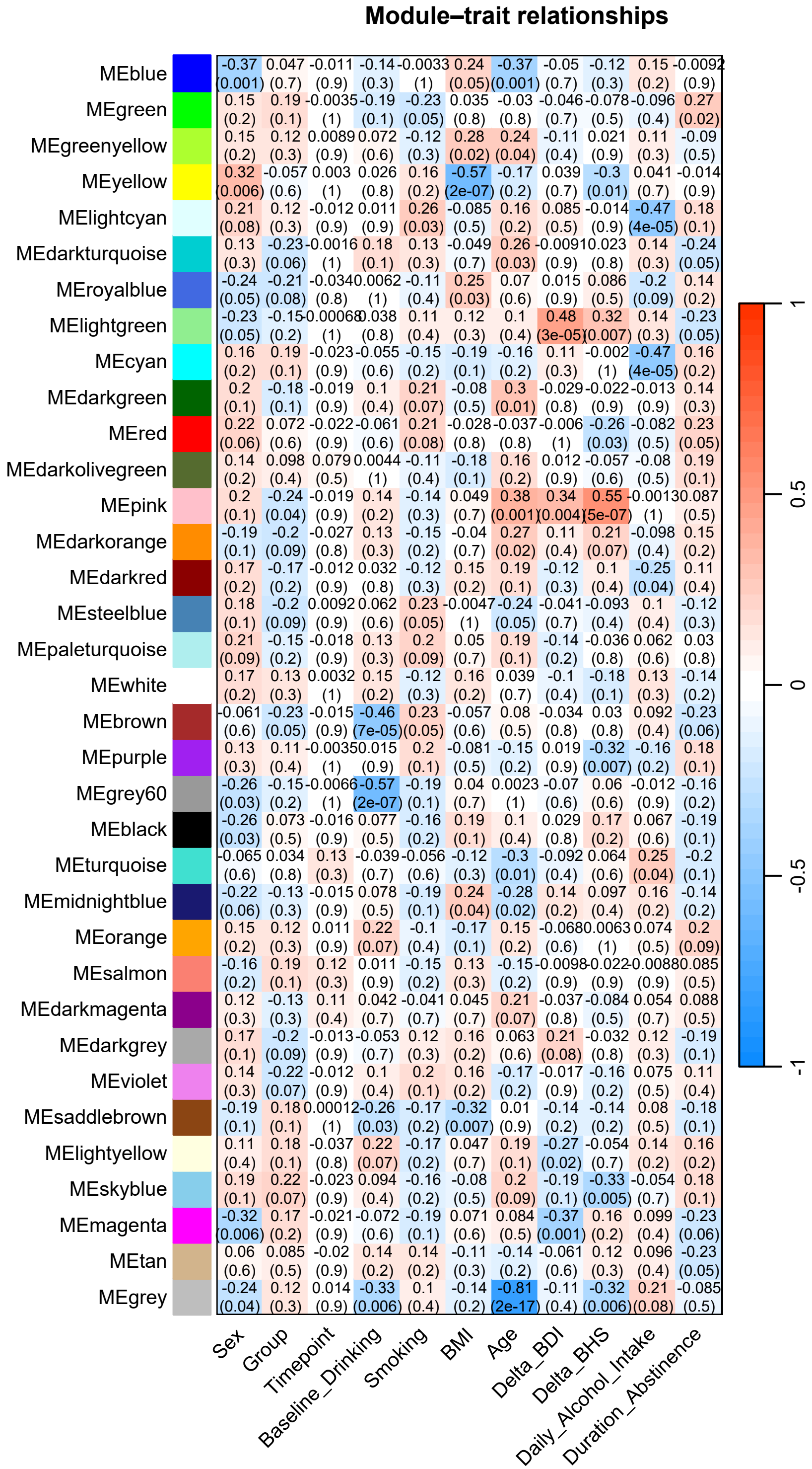

Module–trait relationships
MEblue -0.37 (0.001) 0.047 (0.7) -0.011 (0.9) -0.14 (0.3) -0.0033 (1) 0.24 (0.05) -0.37 (0.001) -0.05 (0.7) -0.12 (0.3) 0.15 (0.2) -0.0092 (0.9)
MEgreen 0.15 (0.2) 0.19 (0.1) -0.0035 (1) -0.19 (0.1) -0.23 (0.05) 0.035 (0.8) -0.03 (0.8) -0.046 (0.7) -0.078 (0.5) -0.096 (0.4) 0.27 (0.02)
MEgreenyellow 0.15 (0.2) 0.12 (0.3) 0.0089 (0.9) 0.072 (0.6) -0.12 (0.3) 0.28 (0.02) 0.24 (0.04) -0.11 (0.4) 0.021 (0.9) 0.11 (0.3) -0.09 (0.5)
MEyellow 0.32 (0.006) -0.057 (0.6) 0.003 (1) 0.026 (0.8) 0.16 (0.2) -0.57 (2e-07) -0.17 (0.2) 0.039 (0.7) -0.3 (0.01) 0.041 (0.7) -0.014 (0.9)
MElightcyan 0.21 (0.08) 0.12 (0.3) -0.012 (0.9) 0.011 (0.9) 0.26 (0.03) -0.085 (0.5) 0.16 (0.2) 0.085 (0.5) -0.014 (0.9) -0.47 (4e-05) 0.18 (0.1)
MEdarkturquoise 0.13 (0.3) -0.23 (0.06) -0.0016 (1) 0.18 (0.1) 0.13 (0.3) -0.049 (0.7) 0.26 (0.03) -0.0091 (0.9) 0.023 (0.8) 0.14 (0.3) -0.24 (0.05)
MEroyalblue -0.24 (0.05) -0.21 (0.08) -0.034 (0.8) 0.0062 (1) -0.11 (0.4) 0.25 (0.03) 0.07 (0.6) 0.015 (0.9) 0.086 (0.5) -0.2 (0.09) 0.14 (0.2)
MElightgreen -0.23 (0.05) -0.15 (0.2) -0.00068 (1) 0.038 (0.8) 0.11 (0.4) 0.12 (0.3) 0.1 (0.4) 0.48 (3e-05) 0.32 (0.007) 0.14 (0.3) -0.23 (0.05)
MEcyan 0.16 (0.2) 0.19 (0.1) -0.023 (0.9) -0.055 (0.6) -0.15 (0.2) -0.19 (0.1) -0.16 (0.2) 0.11 (0.3) -0.002 (1) -0.47 (4e-05) 0.16 (0.2)
MEdarkgreen 0.2 (0.1) -0.18 (0.1) -0.019 (0.9) 0.1 (0.4) 0.21 (0.07) -0.08 (0.5) 0.3 (0.01) -0.029 (0.8) -0.022 (0.9) -0.013 (0.9) 0.14 (0.3)
MEred 0.22 (0.06) 0.072 (0.6) -0.022 (0.9) -0.061 (0.6) 0.21 (0.08) -0.028 (0.8) -0.037 (0.8) -0.006 (1) -0.26 (0.03) -0.082 (0.5) 0.23 (0.05)
MEdarkolivegreen 0.14 (0.2) 0.098 (0.4) 0.079 (0.5) 0.0044 (1) -0.11 (0.4) -0.18 (0.1) 0.16 (0.2) 0.012 (0.9) -0.057 (0.6) -0.08 (0.5) 0.19 (0.1)
MEpink 0.2 (0.1) -0.24 (0.04) -0.019 (0.9) 0.14 (0.2) -0.14 (0.3) 0.049 (0.7) 0.38 (0.001) 0.34 (0.004) 0.55 (5e-07) -0.0013 (1) 0.087 (0.5)
MEdarkorange -0.19 (0.1) -0.2 (0.09) -0.027 (0.8) 0.13 (0.3) -0.15 (0.2) -0.04 (0.7) 0.27 (0.02) 0.11 (0.4) 0.21 (0.07) -0.098 (0.4) 0.15 (0.2)
MEdarkred 0.17 (0.2) -0.17 (0.2) -0.012 (0.9) 0.032 (0.8) -0.12 (0.3) 0.15 (0.2) 0.19 (0.1) -0.12 (0.3) 0.1 (0.4) -0.25 (0.04) 0.11 (0.4)
MEsteelblue 0.18 (0.1) -0.2 (0.09) 0.0092 (0.9) 0.062 (0.6) 0.23 (0.05) -0.0047 (1) -0.24 (0.05) -0.041 (0.7) -0.093 (0.4) 0.1 (0.4) -0.12 (0.3)
MEpaleturquoise 0.21 (0.09) -0.15 (0.2) -0.018 (0.9) 0.13 (0.3) 0.2 (0.09) 0.05 (0.7) 0.19 (0.1) -0.14 (0.2) -0.036 (0.8) 0.062 (0.6) 0.03 (0.8)
MEwhite 0.17 (0.2) 0.13 (0.3) 0.0032 (1) 0.15 (0.2) -0.12 (0.3) 0.16 (0.2) 0.039 (0.7) -0.1 (0.4) -0.18 (0.1) 0.13 (0.3) -0.14 (0.2)
MEbrown -0.061 (0.6) -0.23 (0.05) -0.015 (0.9) -0.46 (7e-05) 0.23 (0.05) -0.057 (0.6) 0.08 (0.5) -0.034 (0.8) 0.03 (0.8) 0.092 (0.4) -0.23 (0.06)
MEpurple 0.13 (0.3) 0.11 (0.4) -0.0035 (1) 0.015 (0.9) 0.2 (0.1) -0.081 (0.5) -0.15 (0.2) 0.019 (0.9) -0.32 (0.007) -0.16 (0.2) 0.18 (0.1)
MEgrey60 -0.26 (0.03) -0.15 (0.2) -0.0066 (1) -0.57 (2e-07) -0.19 (0.1) 0.04 (0.7) 0.0023 (1) -0.07 (0.6) 0.06 (0.6) -0.012 (0.9) -0.16 (0.2)
MEblack -0.26 (0.03) 0.073 (0.5) -0.016 (0.9) 0.077 (0.5) -0.16 (0.2) 0.19 (0.1) 0.1 (0.4) 0.029 (0.8) 0.17 (0.2) 0.067 (0.6) -0.19 (0.1)
MEturquoise -0.065 (0.6) 0.034 (0.8) 0.13 (0.3) -0.039 (0.7) -0.056 (0.6) -0.12 (0.3) -0.3 (0.01) -0.092 (0.4) 0.064 (0.6) 0.25 (0.04) -0.2 (0.1)
MEmidnightblue -0.22 (0.06) -0.13 (0.3) -0.015 (0.9) 0.078 (0.5) -0.19 (0.1) 0.24 (0.04) -0.28 (0.02) 0.14 (0.2) 0.097 (0.4) 0.16 (0.2) -0.14 (0.2)
MEorange 0.15 (0.2) 0.12 (0.3) 0.011 (0.9) 0.22 (0.07) -0.1 (0.4) -0.17 (0.1) 0.15 (0.2) -0.068 (0.6) 0.0063 (1) 0.074 (0.5) 0.2 (0.09)
MEsalmon -0.16 (0.2) 0.19 (0.1) 0.12 (0.3) 0.011 (0.9) -0.15 (0.2) 0.13 (0.3) -0.15 (0.2) -0.0098 (0.9) -0.022 (0.9) -0.0088 (0.9) 0.085 (0.5)
MEdarkmagenta 0.12 (0.3) -0.13 (0.3) 0.11 (0.4) 0.042 (0.7) -0.041 (0.7) 0.045 (0.7) 0.21 (0.07) -0.037 (0.8) -0.084 (0.5) 0.054 (0.7) 0.088 (0.5)
MEdarkgrey 0.17 (0.1) -0.2 (0.09) -0.013 (0.9) -0.053 (0.7) 0.12 (0.3) 0.16 (0.2) 0.063 (0.6) 0.21 (0.08) -0.032 (0.8) 0.12 (0.3) -0.19 (0.1)
MEviolet 0.14 (0.3) -0.22 (0.07) -0.012 (0.9) 0.1 (0.4) 0.2 (0.1) 0.16 (0.2) -0.17 (0.2) -0.017 (0.9) -0.16 (0.2) 0.075 (0.5) 0.11 (0.4)
MEsaddlebrown -0.19 (0.1) 0.18 (0.1) 0.00012 (1) -0.26 (0.03) -0.17 (0.2) -0.32 (0.007) 0.01 (0.9) -0.14 (0.2) -0.14 (0.2) 0.08 (0.5) -0.18 (0.1)
MElightyellow 0.11 (0.4) 0.18 (0.1) -0.037 (0.8) 0.22 (0.07) -0.17 (0.2) 0.047 (0.7) 0.19 (0.1) -0.27 (0.02) -0.054 (0.7) 0.14 (0.2) 0.16 (0.2)
MEskyblue 0.19 (0.1) 0.22 (0.07) -0.023 (0.9) 0.094 (0.4) -0.16 (0.2) -0.08 (0.5) 0.2 (0.09) -0.19 (0.1) -0.33 (0.005) -0.054 (0.7) 0.18 (0.1)
MEmagenta -0.32 (0.006) 0.17 (0.2) -0.021 (0.9) -0.072 (0.6) -0.19 (0.1) 0.071 (0.6) 0.084 (0.5) -0.37 (0.001) 0.16 (0.2) 0.099 (0.4) -0.23 (0.06)
MEtan 0.06 (0.6) 0.085 (0.5) -0.02 (0.9) 0.14 (0.2) 0.14 (0.2) -0.11 (0.3) -0.14 (0.2) -0.061 (0.6) 0.12 (0.3) 0.096 (0.4) -0.23 (0.05)
MEgrey -0.24 (0.04) 0.12 (0.3) 0.014 (0.9) -0.33 (0.006) 0.1 (0.4) -0.14 (0.2) -0.81 (2e-17) -0.11 (0.4) -0.32 (0.006) 0.21 (0.08) -0.085 (0.5)
Sex
Group
Timepoint
Baseline_Drinking
Smoking
BMI
Age
Delta_BDI
Delta_BHS
Daily_Alcohol_Intake
Duration_Abstinence
1
0.5
0
-0.5
-1
