# Supplementary material for: Epigenome-wide association study of psilocybin-induced methylome changes in alcohol use disorder
Source: Transl Psychiatry. 2026 May 26;16:283. doi: 10.1038/s41398-026-03961-3 (PMC13212986; doi:10.1038/s41398-026-03961-3)
Supplement: Supplementary file 6 — Supplementary Figure 5 [file 41398_2026_3961_MOESM6_ESM.pdf]

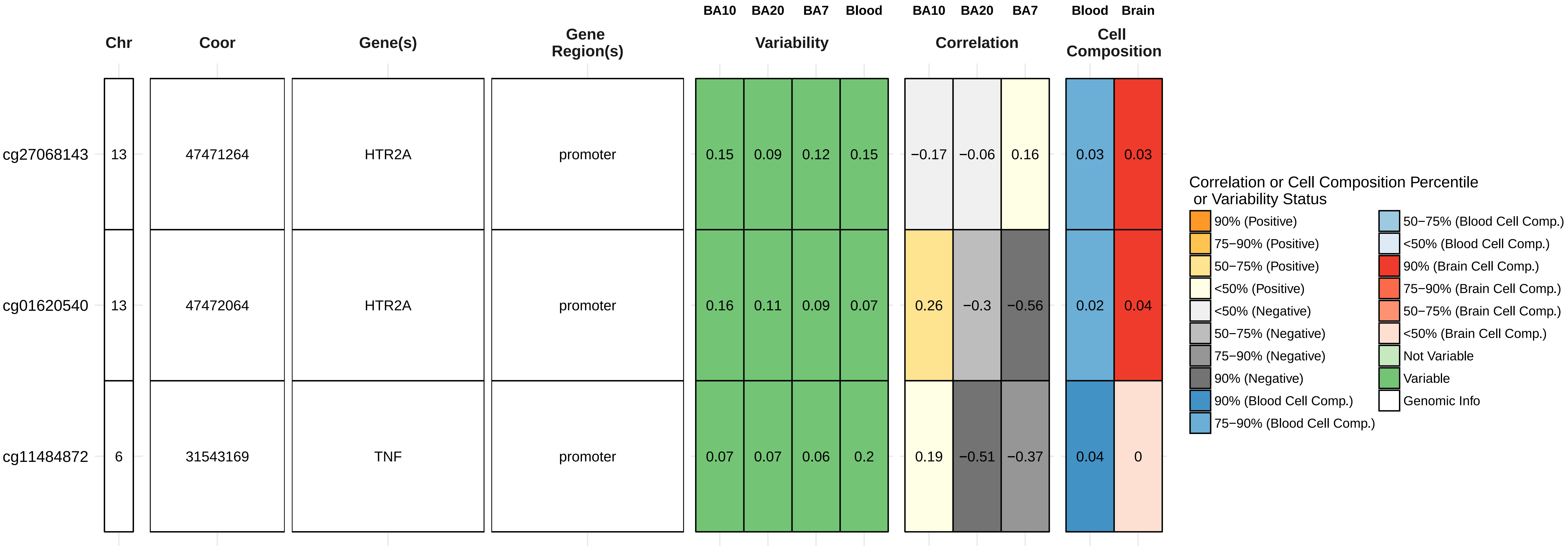

Chr
Coor
Gene(s)
Gene Region(s)
BA10 BA20 BA7 Blood
Variability
BA10 BA20 BA7
Correlation
Blood Brain
Cell Composition
cg27068143 13 47471264 HTR2A promoter 0.15 0.09 0.12 0.15 −0.17 −0.06 0.16 0.03 0.03
cg01620540 13 47472064 HTR2A promoter 0.16 0.11 0.09 0.07 0.26 −0.3 −0.56 0.02 0.04
cg11484872 6 31543169 TNF promoter 0.07 0.07 0.06 0.2 0.19 −0.51 −0.37 0.04 0
Correlation or Cell Composition Percentile or Variability Status
90% (Positive)
75−90% (Positive)
50−75% (Positive)
<50% (Positive)
<50% (Negative)
50−75% (Negative)
75−90% (Negative)
90% (Negative)
90% (Blood Cell Comp.)
75−90% (Blood Cell Comp.)
50−75% (Blood Cell Comp.)
<50% (Blood Cell Comp.)
90% (Brain Cell Comp.)
75−90% (Brain Cell Comp.)
50−75% (Brain Cell Comp.)
<50% (Brain Cell Comp.)
Not Variable
Variable
Genomic Info
